# Isolation of Bacterial Ribosomes with Monolith Chromatography

**DOI:** 10.1371/journal.pone.0016273

**Published:** 2011-02-04

**Authors:** Andrej Trauner, Mark H. Bennett, Huw D. Williams

**Affiliations:** Department of Life Sciences, Imperial College London, London, United Kingdom; University of South Florida College of Medicine, United States of America

## Abstract

We report the development of a rapid chromatographic method for the isolation of bacterial ribosomes from crude cell lysates in less than ten minutes. Our separation is based on the use of strong anion exchange monolithic columns. Using a simple stepwise elution program we were able to purify ribosomes whose composition is comparable to those isolated by sucrose gradient ultracentrifugation, as confirmed by quantitative proteomic analysis (iTRAQ). The speed and simplicity of this approach could accelerate the study of many different aspects of ribosomal biology.

## Introduction

The translating bacterial ribosome, comprising more than 50 proteins, 3 ribosomal RNAs, mRNA and tRNA, is among the more complex structures in the prokaryotic cell [1]. Ribosomes are central to cellular function – a fact very clearly illustrated by the number of antibiotics, which target their function. The complexity of their composition, structure and function requires the implementation of a range of analytical techniques and almost all of them rely on the isolation of ribosomes using density gradient centrifugation, which is the gold standard for purifying ribosomes prior to further analyses [1], [2], [3], [4]. However, it is a lengthy and labour intensive procedure. The proteomic study of growth-phase dependent as well as environmental stress induced changes in prokaryotic ribosomes and their associated factors has been hindered by the absence of a fast and efficient purification method. Chromatography has been used in the past in an attempt to accelerate and simplify the isolation process [5], [6], [7], [8], [9]. While such methods never became widely used, there has been a recent renewal of interest in improving the potential of chromatography for isolating ribosomes [10]. Furthermore, advances in genetic manipulation tools have allowed affinity purification to be applied to ribosome isolation, with good results [11], [12], [13]. Each of these approaches has its merits; however the speed of separation is always inherently limited by the architecture of the chromatographic matrix. High backpressures caused by the size of ribosomes severely limit the maximum flow rate that can be attained, thus greatly increasing the overall time taken to obtain ribosomal fractions. There is scope to develop a robust, universal, rapid and easy way to isolate ribosomes using chromatography. Monolith columns are a new class of chromatographic stationary phase, based on a highly cross-linked porous monolithic polymer. Unlike conventional chromatography columns packed with porous particles, the monolithic column is a single piece of porous structure of uninterrupted and interconnected channels. The sample is transported through the column via convection leading to very fast mass transfer between the mobile and stationary phase even for large biomolecules [14]. The absence of matrix packing leads to low backpressures allowing high flow rates to be achieved, leading to rapid separations even for very large biomolecules such as protein complexes, immunoglobulins and viruses [15], [16]. Consequently, we decided to investigate whether monolithic chromatography would be suitable for rapid purification of bacterial ribosomes, and as we have an interest in the composition of mycobacterial ribosome, we used *Mycobacterium smegmatis* as the model for these studies.

Here we report an accessible method, based on monolithic columns, that allows the isolation of salt-washed ribosomes from crude cellular extracts of different bacteria in less than 10 minutes.

## Results

### Ribosomal chromatography

**Figure 1:**
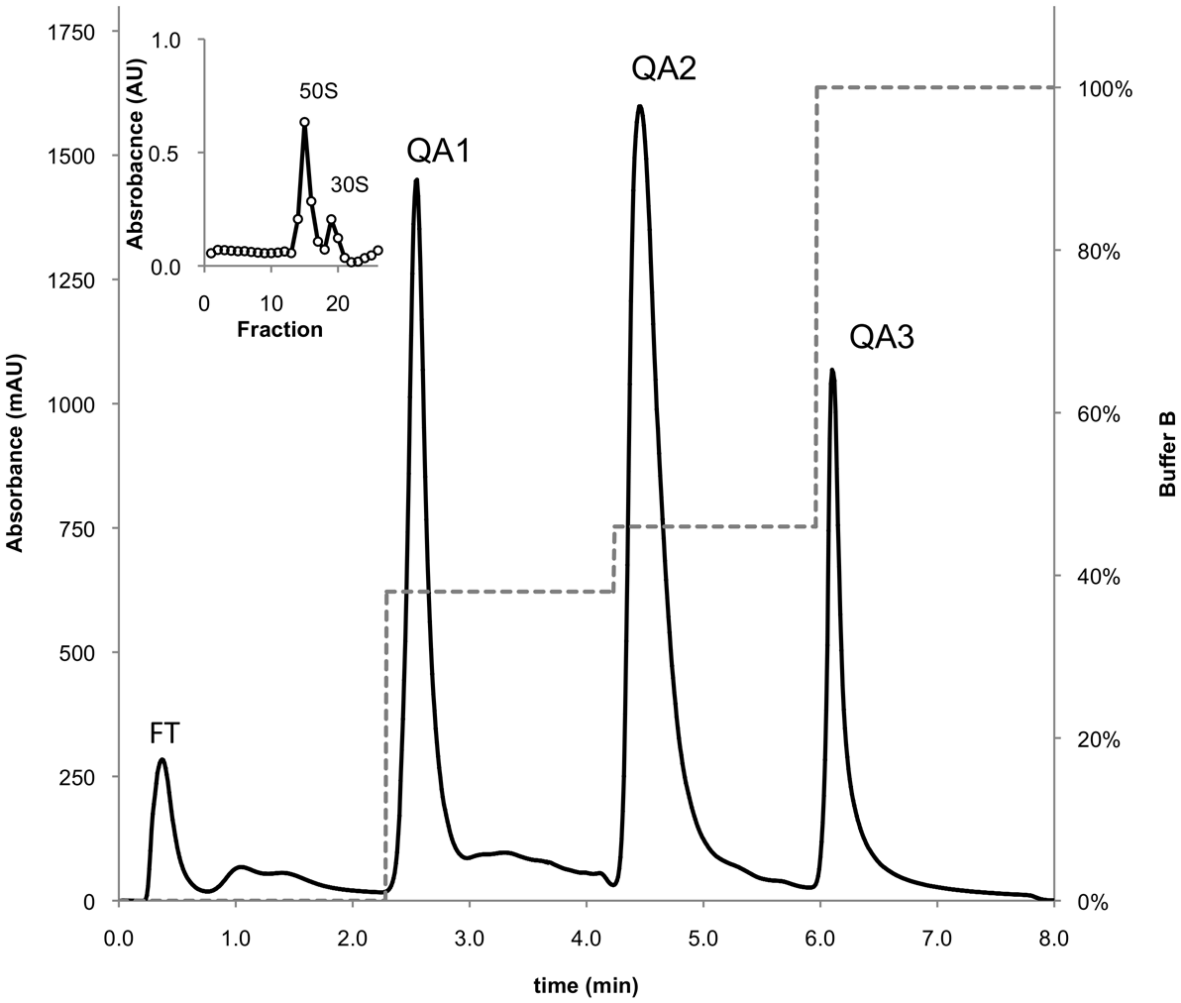
Purification of *M. smegmatis* ribosomes using monolithic columns. M. smegmatis cell extracts were loaded onto a quaternary amine monolithic column and ribosomes were isolated following a stepwise elution. The absorbance (solid line) and the proportion of Buffer B (dashed line) are shown and the flow-through (FT) as well as fractions QA1-3 are annotated. Fractions from QA2 were analysed by linear sucrose gradient ultracentrifugation and found to contain 30S and 50S ribosomal subunits (inset).

**Figure 2:**
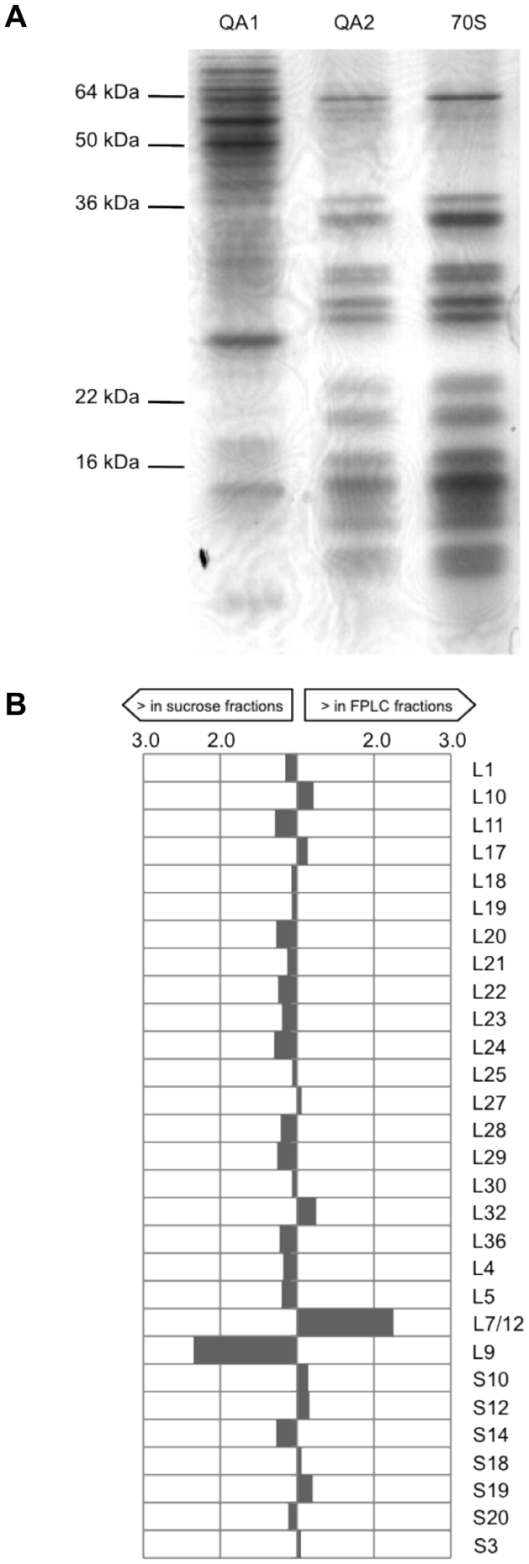
Chromatographic fraction QA2 and sucrose-purified ribosomes contain comparable levels of ribosomal proteins. (**A**) Proteins from QA1 and QA2 were precipitated and compared to sucrose-purified ribosomes (70S) by SDS-PAGE. (**B**) Sucrose-gradient purified ribosomal samples and chromatography-purified ribosomal samples were analysed by iTRAQ coupled to HPLC-MSMS. The relative abundance of ribosomal proteins whose levels varied between the two preparations is shown.

The architecture of monolithic chromatography columns is well suited for the separation of large molecular complexes [17], as illustrated by the ease with which they can be used to isolate intact and active bacteriophages [18]. We were interested to see whether we could devise an analogous method for the purification of bacterial ribosomes. Strong anion exchange (quaternary amine – QA) chemistry was selected, as there are significant areas of exposed negatively charged rRNA on the surface of the ribosome [1]. Our initial attempts, using a linear NaCl gradient to elute the bound material, revealed that bacterial cell lysates could be fractioned into three main components on QA monolithic columns (termed fractions QA1-3). Given that DNA was reported to elute from monolithic columns at 0.6–0.8 M NaCl [19], we tested the possibility that genomic DNA elutes as a single fraction by pre-treating lysates with RNAse-free DNAse. We were thus able to identity fraction QA3 as genomic DNA (See Fig. S1). The chromatographic programme was modified to three stepwise elutions in order to improve the separation of cellular fractions (Fig. 1). We analysed the unbound material as well as QA1-3 by sucrose gradient ultracentrifugation in an attempt to detect the presence of ribosomes. We were successful in identifying 50S and 30S ribosomal subunits in fraction QA2, and no indication of ribosomal material was found in other fractions (Fig. 1, inset and Fig. S2). We also found that replacing 1 M NaCl with 1 M NH_4_Cl in the elution buffer led to the elution of intact 70S ribosomes, as determined by sucrose gradient ultracentrifugation, without affecting the chromatography (Fig. S3A). SDS-PAGE analysis of the cellular fractions revealed that the same complement of proteins as those found in sucrose purified 70S ribosomes was present in QA2 (Fig. 2A). The pattern of proteins present in QA1 was distinct from sucrose purified 70S ribosomes; while no protein bands were observed in QA3 or the flow-through.

We were able to detect biological activity of monolith purified ribosomes using two complementary approaches - an *in vitro* coupled transcription-translation assay as well as the erythromycin binding assay (Fig. S4).

### Mass spectrometric analysis of protein fractions QA1 and QA2

Mass spectrometry was used for a cursory analysis of fractions QA1 and QA2. QA1 was found to contain a number of cytosolic proteins (enolase, adenylate kinase, pyruvate kinase, chaperones, superoxide dismutase), as well as membrane and cell wall associated proteins (e.g Div IVA, fumarate reductase, ATP synthase). Fraction QA2, on the other hand, contained a large number of different ribosomal proteins as well as a few ribosome-associated proteins (e.g. EF-Tu, trigger factor) and some cytosolic proteins such as enolase, glycerol kinase.

In light of these findings, we implemented quantitative proteomics using mass spectrometry of iTRAQ labelled peptides to compare the protein composition of ribosomes isolated by FPLC-monolith chromatography (fraction QA2) to those isolated by sucrose gradient ultracentrifugation. This approach also enabled us to assess the reproducibility of the chromatographic purification of ribosomes.

As shown in Table 1, we were able to detect the same complement of 50 out of the 53 ribosomal proteins encoded in the *Msm* genome [20] in ribosomes isolated by both, monolith chromatography and sucrose gradient purification. The vast majority of ribosomal proteins were found to be equally as abundant in each of the preparations. The exceptions to this are highlighted in Table 1 and Fig. 2A, the most notable of which (2.2 fold difference) were ribosomal proteins L9 and L7/12, the former was found to be more abundant in sucrose-purified fractions, while the latter was more abundant in chromatographically purified fractions. Furthermore, no significant differences were observed when comparing repeat FPLC runs and/or repeat samples, pointing to the high reproducibility of our technique (Fig. S5). It is important to note that ribosomal proteins L31, L34 and L35 were not detected during our analyses of either chromatographically or sucrose purified ribosomes. We believe that the lack of these proteins in our dataset does not reflect their absence from isolated ribosomes, but points to the technical limitation of the mass spectrometer in detecting these particular proteins [2]. Therefore, we conclude that the protein composition of monolith chromatography purified ribosomes is qualitatively and quantitatively similar to sucrose–gradient purified ribosomes. In addition to ribosomal proteins we were able to identify a number of proteins that do not form an integral part of the ribosome. These could be divided into two groups, proteins that are known to associate to the ribosome (EF-Tu, Trigger factor) and those that are not (Glycerol kinase, Glutamate synthase). More proteins that are not normally considered to be ribosome-associated were present in the monolith chromatography preparations. As some of these proteins have been found to be present in ribosomal fractions during previous studies [2], [13] it may suggest that chromatographically isolated ribosomes are less pure, and the presence of certain proteins just reflects their relative abundance in the cytosol. Alternatively, it may indicate that chromatographically isolated ribosomes retain weakly associated proteins that are lost during sucrose gradient purification making them more suitable for the discovery of transiently or condition-specific ribosome-associated proteins.

**Table 1:** Comparative iTRAQ analysis of *M. smegmatis* ribosomal fractions by mass spectrometry.

Large Subunit Proteins
L1, L2, L3, L4, **L5** a, L6, **L7/12** b, **L9** a, L10, **L11** a, L13, L14, L15, L16, L17, L18, L19, L20, L21, **L22** a, L23, L24, L25, L27, **L28** a, L29, L30, L32, L33, L36
Small Subunit Proteins
S1, S2, S3, S4, S5, S6, S7, S8, S9, S10, S11, S12, S13, S14, S15, S16, S17, S18, S19, S20
Ribosome associated proteins
Elongation factor – Tu, Trigger factor^b^
Non-ribosomal Proteins
Enolase^b^, RNA polymerase^b^, Glutamine synthase^b^, Glycerol kinase^b^, Acyl carrier protein^b^, trypsin

a significantly (p<0.05) more abundant in sucrose purified ribosomal fractions.

b significantly (p<0.05) more abundant in FPLC purified ribosomal fractions.

Despite these differences, overall, the data indicate that the composition of ribosomes obtained by the two approaches is comparable.

## Discussion

We were successful in developing a new, very rapid chromatographic method for the purification of ribosomes based on the use of strong anion exchange monolith chromatography. The method successfully purified 30S and 50S ribosomal subunits and could be modified through switching elution buffers to isolate intact 70S ribosomes. Speed of isolation is both important in allowing large numbers of samples to be processed in a short time period and also in minimising the contact between ribosomes and proteases and nucleases in the cell extract. With our method, from loading the cell lysate onto the column to obtaining pure ribosomes takes as little as 5 minutes – compared to 8 hours using sucrose gradient ultracentrifugation. We envisage that this technique could be exploited for compositional analysis, as it would provide an efficient and rapid tool for the isolation of ribosomes from multiple samples. The scope of this approach could be broadened by combining it with chemical cross-linking, as we do not believe that modifications incurred by such procedures would adversely affect the chromatographic separation. Furthermore, ribosomes are being investigated as possible vaccines [21],[22]. Our method is well suited for such applications and could significantly increase the throughput of such studies.

### Conclusions

The main advantage of our approach is its speed: ribosomal fractions can be obtained from crude extracts in as little as five minutes. It is also very easy to implement, since there is no need for extensive testing or expensive equipment: commercially available columns can be connected to any low-pressure liquid chromatography system, which are almost ubiquitous in modern laboratories, and ribosomes can be obtained using a simple stepwise elution program. When testing the method, we were able to purify *Escherichia coli* ribosomes using the same conditions (data not shown); if QA interacting with the phosphate backbone of nucleic acids is indeed the basis of separation, we see no reason why this technique could not be applicable to a broad spectrum of organisms. Finally, we have performed over two hundred runs over the past two years using the same columns, without observing any deterioration of the separation.

## Materials and Methods

### Reagents

Yeast extract and tryptone were obtained from Oxoid, Basingstoke, UK. RNAse-free DNAse was obtained from New England Biolabs, Ipswich, MA, USA. Trypsin and the iTRAQ labelling kit were obtained from Applied Biosystems, Foster City, CA, USA. All other chemicals were of analytical grade purity and were obtained from Sigma-Aldrich, Gillingham, UK.

### Bacterial strains and growth media

*Mycobacterium smegmatis* strain MC^2^155 (*Msm*) was grown in LB medium (5 g yeast extract, 5 g NaCl, 10 g Tryptone per litre) with continuous shaking (200 rpm) at 37°C. 0.04% (*v/v*) Tween 80 was added to LB to avoid clumping of *Msm*.

### Preparation of cell lysates

Cells were grown to mid-exponential phase (OD_600_ ≈0.8) and harvested by centrifugation (10 min at 10,000×*g*, 4°C). The cell pellet was washed in lysis buffer (70 mM KCl, 10 mM MgCl_2_, 10 mM Tris-HCl [pH 7.4]) and frozen at −80°C. When needed the cells were thawed, resuspended in lysis buffer (1.0 g_wet weight_ ml^−1^) and broken in a French press (10,000 psi). The lysate was clarified by centrifugation (60 min at 30,000×*g*, 4°C); the pellet containing unbroken cells and cellular debris was discarded.

### Chromatographic purification of ribosomes

Cell lysates were diluted in lysis buffer and filtered through a 0.22 µm filter (Sartorius, Epsom, UK) prior to fast protein liquid chromatography (FPLC). 0.5 ml of the filtered sample (1.5–4.5 mg ml^−1^) was injected into an ÄktaFPLC system (GE Healthcare) and loaded onto two quaternary amine Convective Interaction Media (QA) monolithic discs (Total column volume 0.68 ml, BIA Separations) encased within a polyoxomethylene (POM) casing (BIA Separations), which have been equilibrated with lysis buffer. All chromatography was performed at a flow rate of 2 ml min^−1^ using lysis buffer (A) and lysis buffer + 1M NaCl (B) as the mobile phases. The column was washed with 7 column volumes buffer A and eluted sequentially with 38%, 46% and 100% buffer B. The resulting fractions containing unbound material (flow through) as well as molecules eluted at each step were collected for further analysis.

### Coupled transcription-translation assay

The assay was based on the commercially available “*E. coli* S30 Extract System for Circular DNA” kit (Promega). The standard protocol specified by the manufacturer was modified for the coupled transcription-translation in the following way: ribosomes from the S30 extract were pelleted by centrifugation - 2 h at 31,000 rpm at 4°C, in a Beckman MLS-50 rotor (Beckman-Coulter) using an Optima Max tabletop ultracentrifuge (Beckman-Coulter). The supernatant was carefully removed (S100 extract) and added to FPLC purified ribosomes. Two types of purified ribosome preparations were used: we either used an aliquot of the ribosome containing fraction eluted from the QA column, or we concentrated the eluted ribosomes by centrifugation - 2 h at 31,000 rpm at 4°C, in a Beckman MLS-50 rotor (Beckman-Coulter) using an Optima Max tabletop ultracentrifuge (Beckman-Coulter) and resuspended them in ribosomal buffer prior to the assay. Purified ribosomes were supplemented with a complete amino acid mix, the appropriate amount of the “Premix” supplied with the kit and an appropriate amount of the S100 extract. Their ability to produce firefly luciferase was compared to that of the luciferase control, which was synthesised as specified by the manufacturer.

Bioluminescence of the synthesised firefly luciferase was measured by adding 20 µl of Luciferase Assay Reagent (Promega) to the assay mixtures that have been adjusted to a final volume of 1 ml in phosphate-buffered saline. Raw data were collected in duplicate over a period of 30 s using a Berthold AutoLumat LB953 tube luminometer.

### [^3^H]-Erythromycin binding assay

The assay was carried using a modification of the protocol described by Maguire *et al.* [10]. Briefly, FPLC purified ribosomes were concentrated by using 3,000 Da molecular size cut-off centrifugation filters (Amicon) to a final volume of 100 µl. The ribosomes were resuspended in 10 ml of lysis buffer and concentrated again using the size exclusion filter to 100 µl. 10 µl aliquots of ribosomes were incubated for 15 minutes with 1, 0.5, 0.2 or 0.1 µCi of [N-methyl-^3^H]-erythromycin (American Radiolabelled Chemicals). After the incubation the reaction mixture was filtered onto a glass fibre disc (GF/C, φ13 mm, Millipore). Unbound [^3^H]-erythromycin was washed off with 5 ml of ethanol. The ribosome bound radiolabel was determined by transferring the washed filter into a scintillation vial with 3 ml of UltimaGold scintillation liquid (Perkin-Elmer) and measured using a Wallac scintillation counter (Perkin-Elmer). Raw data were collected in duplicate over a period of 5 min using the pre-set parameters for ^3^H.

### Sucrose gradient purification of ribosomes

Linear sucrose density gradients (15–40%) were prepared by layering 5.5 ml of a 15% sucrose solution (15% [*w*/*v*] sucrose, 50 mM Tris-HCl [pH 7.4], 25 mM KCl, 10 mM MgCl_2_) on top of 5.5 ml of a 40% sucrose solution (40% [*w*/*v*] sucrose, 50 mM Tris-HCl [pH 7.4], 25 mM KCl, 10 mM MgCl_2_) in Polyallomer thin walled ultracentrifuge tubes (Beckman-Coulter). The tubes were sealed with Parafilm M (Pechiney Plastic Packaging Company), rotated gently to a horizontal position and left at room temperature for 2.5 h to allow the gradient to form by diffusion. 200 µl of clarified cell lysate was incubated with RNase-free DNase for 1 h and then centrifuged through a sucrose cushion (1.1 M sucrose, 50 mM Tris-HCl [pH 7.4], 25 mM KCl, 10 mM MgCl2) for 1.5 h at 31,000 rpm and 4°C in a Beckman MLS-50 rotor (Beckman-Coulter) using an Optima Max tabletop ultracentrifuge (Beckman-Coulter). The supernatant was discarded, while the ribosomal pellet was gently resuspended in lysis buffer, loaded onto the linear sucrose gradient and centrifuged (5 h at 35,000 rpm, 4°C) in a SW 41-Ti Rotor (Beckman-Coulter) using an Optima L-100 XP ultracentrifuge (Beckman-Coulter). The gradients were subsequently fractionated into 400 µl fractions and their absorbance at 254 nm, 260 nm and 280 nm measured using a DU640 spectrophotometer (Beckman-Coulter). Fractions containing 70S ribosomes were pooled and used for further analysis.

### SDS-PAGE analysis of proteins

Proteins were precipitated by the addition of ice-cold acetone (4-times sample volume) and overnight incubation at −20°C, followed by centrifugation (15 min at 16,000×*g*, 4°C). Precipitated proteins were resuspended in 25 µl distilled water and 5 µl of 6x Sample loading buffer (Sigma-Aldrich) was added to each sample. Samples were boiled for 5 min prior to loading onto a 15% polyacrylamide-SDS gel. Gels were run at 150 V for 2 h, stained using Imperial Blue (Thermo) and developed by washing in distilled water.

### iTRAQ labelling of digested ribosomal proteins

Proteins were precipitated as for SDS-PAGE analysis. The pellets were air-dried and resuspended in dissolution buffer provided in the iTRAQ labelling kit (Applied Biosystems). Protein content was quantified using the Coomassie Plus (Thermo) reagent and 100 µg samples were labelled with the isobaric iTRAQ reagent according to the manufacturer's protocol. Briefly, protein samples were reduced with tris-(2-carboxyethyl) phosphine, cysteine-blocked with methyl methanethiosulfonate and digested with trypsin overnight at 37°C. The content of one iTRAQ reagent vial was added to each sample, allowed to react and all samples were combined after labelling. In total we analysed ribosomal peptides isolated from four FPLC purifications and three sucrose gradient ultracentrifugations. To remove chemicals that may interfere with mass spectrometry the peptides were purified using an iCAT Cation Exchange Cartridge (Applied Biosystems). The eluent was vacuum-dried in a SpeedVac, resuspended in 100 µl of distilled water and used for mass spectrometric analysis.

### Peptide analysis using high pressure liquid chromatography and mass spectrometry

Samples were loaded on a Zorbax SB-C18 5 µm, 35×0.5 mm (Agilent) trap column and washed for 60 min using 96.7% water: 3% acetonitrile: 0.3% formic acid; the extended wash was to remove residual KCl remaining from the ion exchange purification step. Peptides were separated using a Zorbax 300SB-C18 5 µm, 150×0.3 mm capillary column (Agilent) at a flow rate of 5 µl min^−1^ using an Agilent 1100 HPLC system. Buffer C (94.9% water: 5% acetonitrile: 0.1% formic acid) and Buffer D (94.9% acetonitrile: 5% water: 0.1% formic acid) were used for the elution of peptides according to the following program: gradient 0–30% Buffer D over 90 min, gradient 30–90% Buffer D over 10 min, 90% Buffer D for 10 min, gradient 90–100% Buffer D over 1 min and 100% Buffer D for 10 min. Eluted peptides were analysed using a Q-TRAP mass spectrometer (Applied Biosystems) equipped with a Turbo Spray Ion source at 150°C. Data were collected with an IDA method consisting of a survey scan (350 m/z to 1200 m/z), an enhanced resolution scan and four enhanced product ion scans. Dynamic background subtraction was used prior to ion selection; the four most abundant doubly or triply charged ions were selected for the product ion scans. The resulting spectra were analysed and quantified using ProteinPilot software (Applied Biosystems), quoted significance values were obtained with the inbuilt statistical analysis tool.

## Supporting Information

Figure S1 **Linear gradient elution of** ***M. smegmatis*** **ribosomes from monolithic columns.** The absorbance trace of a cell lysate sample (gray solid line), DNase treated cell lysate sample (black solid line) and the proportion of Buffer B (dashed line) are shown. Fractions QA1-3 are annotated. NB In these experiments the concentration of NaCl in Buffer B was 1.5 M.
(TIF)

Figure S2 **Linear sucrose gradient ultracentrifugation of chromatographic fractions indicated in** **Figure 1** **.** FT (**A**), QA1 (**B**) and QA3 (**C**). No evidence of ribosomes or ribosomal subunits was detected - ribosomal subunits were found only in fraction QA2 (Fig. 1, inset).
(TIF)

Figure S3 **FPLC purification can yield associated ribosomes and maintains the ratio of subunits in the sample.** (**A**) Fraction QA2 – see Fig. S1 – was eluted with NH_4_Cl collected, ultracentrifuged to pellet ribosomes and used for ribosomal profiling. (**B,C**) The ratios plotted on the abscissa were calculated for peptides identified with a confidence of 95% using ProteinPilot software. The software determines these ratios by dividing the intensity of the signal derived from the label reporter moieties for the samples chosen by the user. We collated the ratios for FPLC purified/sucrose purified peptides for 30S (**B**) and 50S (**C**) subunits.
(TIF)

Figure S4 **Monolith-FPLC purified ribosomes show biological activity.** (**A**) Ribosomes from actively growing *E. coli* were isolated by monolith FPLC and used for coupled transcription-translation (T-T) assay expressing firefly luciferase. “FPLC+” ribosomes isolated by FPLC and concentrated by ultracentrifugation, “FPLC” ribosomes isolated by chromatography, “No DNA” unaltered kit without template, “PBS” dilution buffer alone. (**B**) Binding of [^3^H]-erythromycin by FPLC purified *Msm* wild type ribosomes that were concentrated using centrifuge filtration. Control experiments were performed in the absence of ribosomes (to assess the efficiency of the wash step to remove unbound antibiotic from filters). T-T: histograms represent the average of 10 readings; error bars correspond to the standard deviation (N = 10). [^3^H]-erythromycin binding: histograms represent the average of two readings; error bars correspond to the standard deviation (N = 2)
(TIF)

Figure S5 **The composition of ribosomes is comparable for repeat FPLC runs and/or repeat samples.** Ratios were obtained as described in Fig. S3. FPLC1 and FPLC2 are repeat runs of the same biological sample – actively growing *Msm* wild type. FPLC3 and FPLC4 were obtained from an independent sample of actively growing *Msm* wild type. Ratios for 30S peptides (**A**) and 50S peptides (**B**) are shown.
(TIF)
