# Supplementary figures and images for: Isolation of Bacterial Ribosomes with Monolith Chromatography
Source: PLoS One. 2011 Feb 4;6(2):e16273. doi: 10.1371/journal.pone.0016273 (PMC3033897; doi:10.1371/journal.pone.0016273)

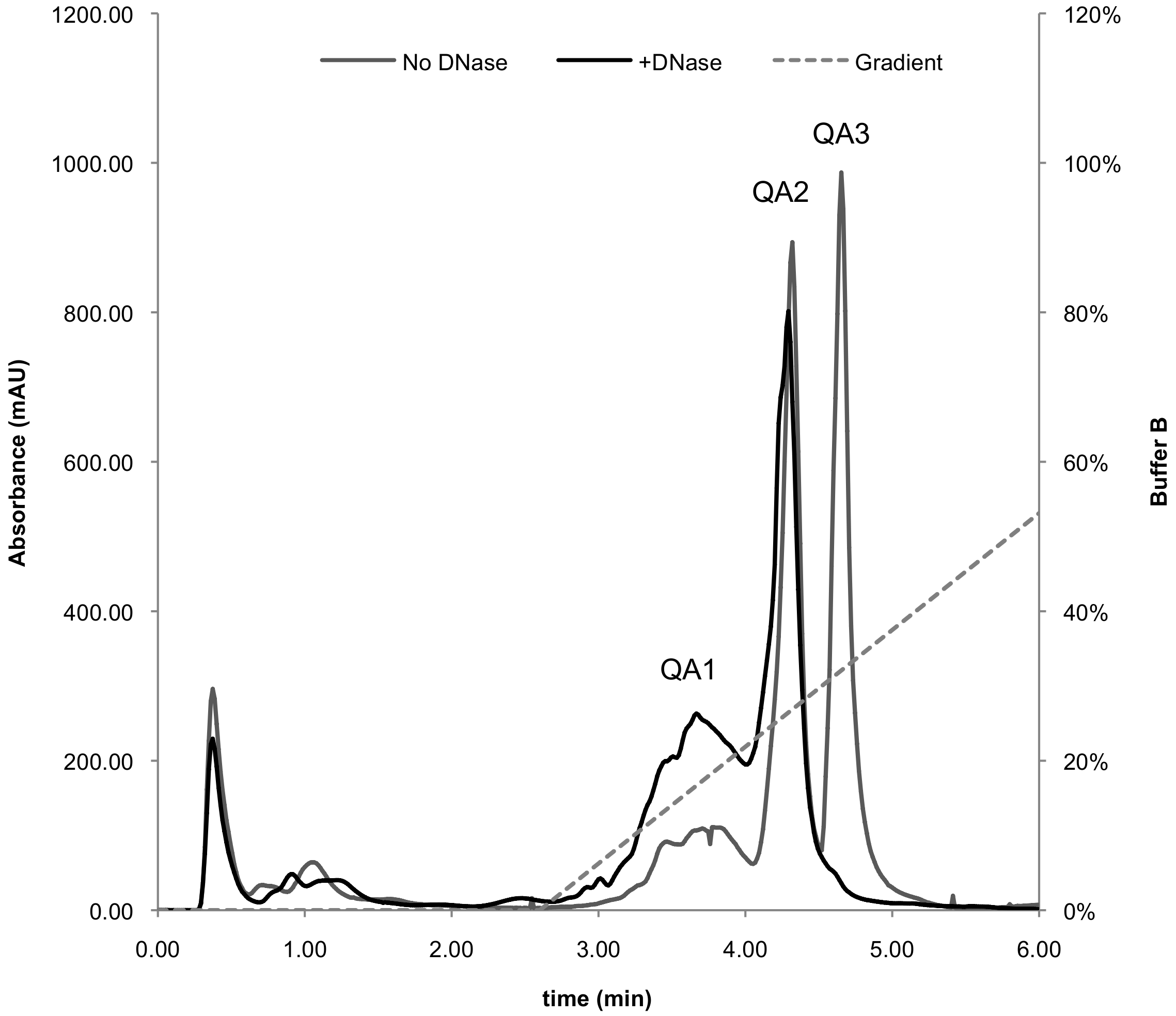

Supplement: Figure S1 — Linear gradient elution of M. smegmatis ribosomes from monolithic columns. The absorbance trace of a cell lysate sample (gray solid line), DNase treated cell lysate sample (black solid line) and the proportion of Buffer B (dashed line) are shown. Fractions QA1-3 are annotated. NB In these experiments the concentration of NaCl in Buffer B was 1.5 M. (TIF) [file pone.0016273.s001.tif]

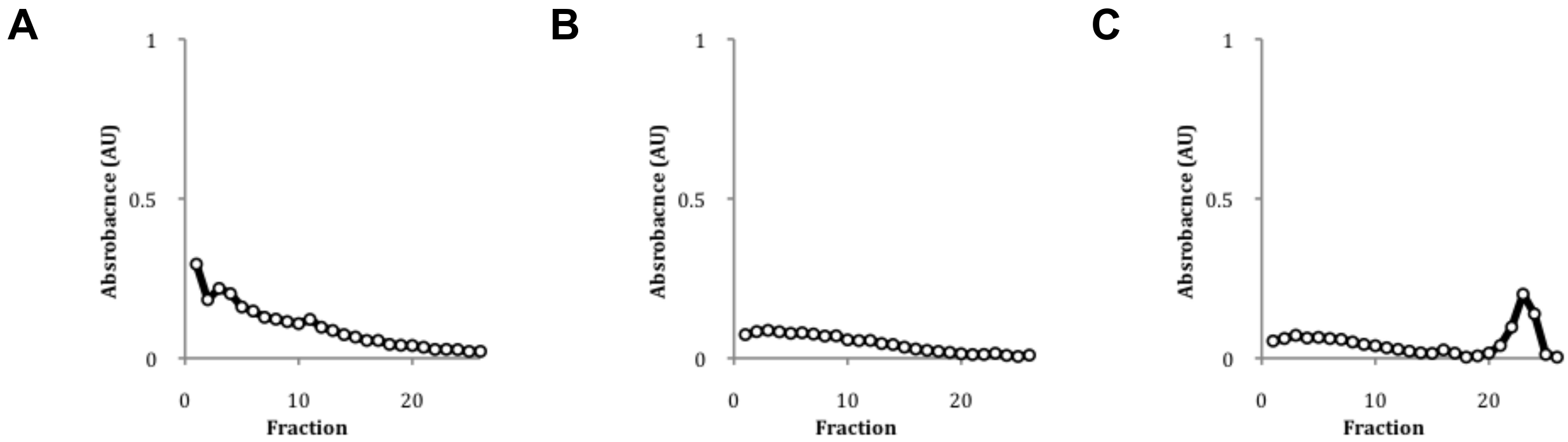

Supplement: Figure S2 — Linear sucrose gradient ultracentrifugation of chromatographic fractions indicated in Figure 1 . FT (A), QA1 (B) and QA3 (C). No evidence of ribosomes or ribosomal subunits was detected - ribosomal subunits were found only in fraction QA2 (Fig. 1, inset). (TIF) [file pone.0016273.s002.tif]

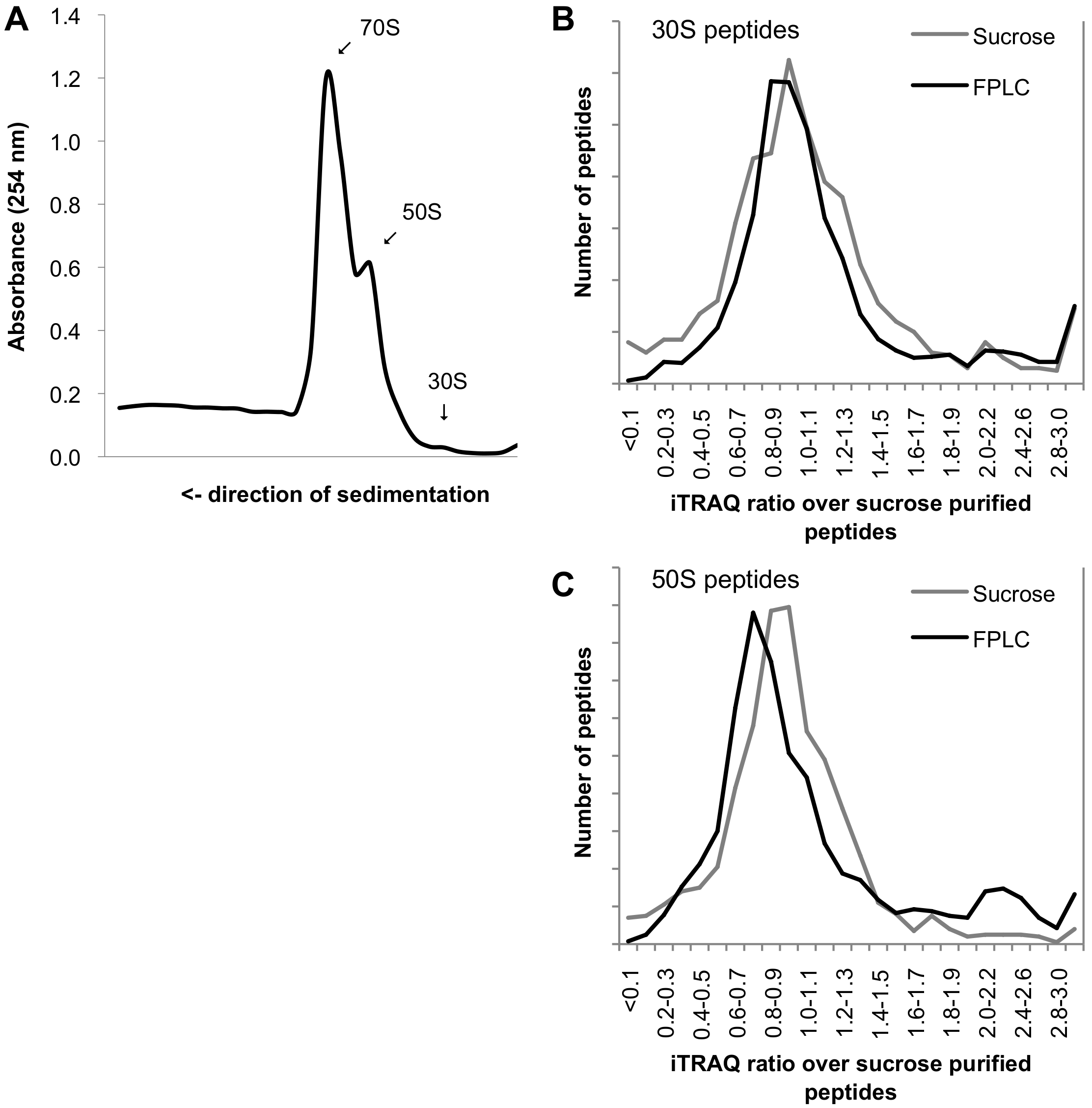

Supplement: Figure S3 — FPLC purification can yield associated ribosomes and maintains the ratio of subunits in the sample. (A) Fraction QA2 – see Fig. S1 – was eluted with NH4Cl collected, ultracentrifuged to pellet ribosomes and used for ribosomal profiling. (B,C) The ratios plotted on the abscissa were calculated for peptides identified with a confidence of 95% using ProteinPilot software. The software determines these ratios by dividing the intensity of the signal derived from the label reporter moieties for the samples chosen by the user. We collated the ratios for FPLC purified/sucrose purified peptides for 30S (B) and 50S (C) subunits. (TIF) [file pone.0016273.s003.tif]

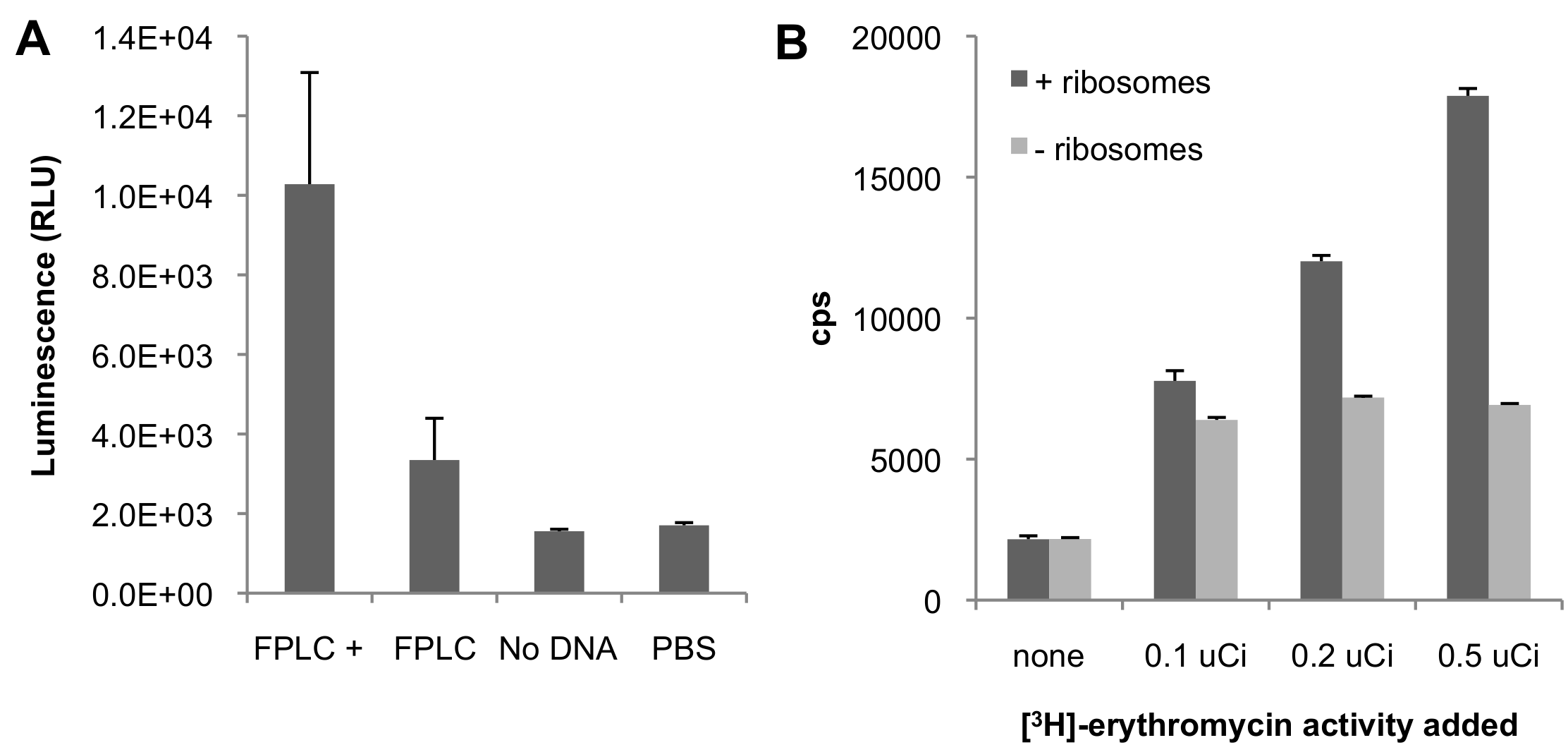

Supplement: Figure S4 — Monolith-FPLC purified ribosomes show biological activity. (A) Ribosomes from actively growing E. coli were isolated by monolith FPLC and used for coupled transcription-translation (T-T) assay expressing firefly luciferase. “FPLC+” ribosomes isolated by FPLC and concentrated by ultracentrifugation, “FPLC” ribosomes isolated by chromatography, “No DNA” unaltered kit without template, “PBS” dilution buffer alone. (B) Binding of [3H]-erythromycin by FPLC purified Msm wild type ribosomes that were concentrated using centrifuge filtration. Control experiments were performed in the absence of ribosomes (to assess the efficiency of the wash step to remove unbound antibiotic from filters). T-T: histograms represent the average of 10 readings; error bars correspond to the standard deviation (N = 10). [3H]-erythromycin binding: histograms represent the average of two readings; error bars correspond to the standard deviation (N = 2) (TIF) [file pone.0016273.s004.tif]

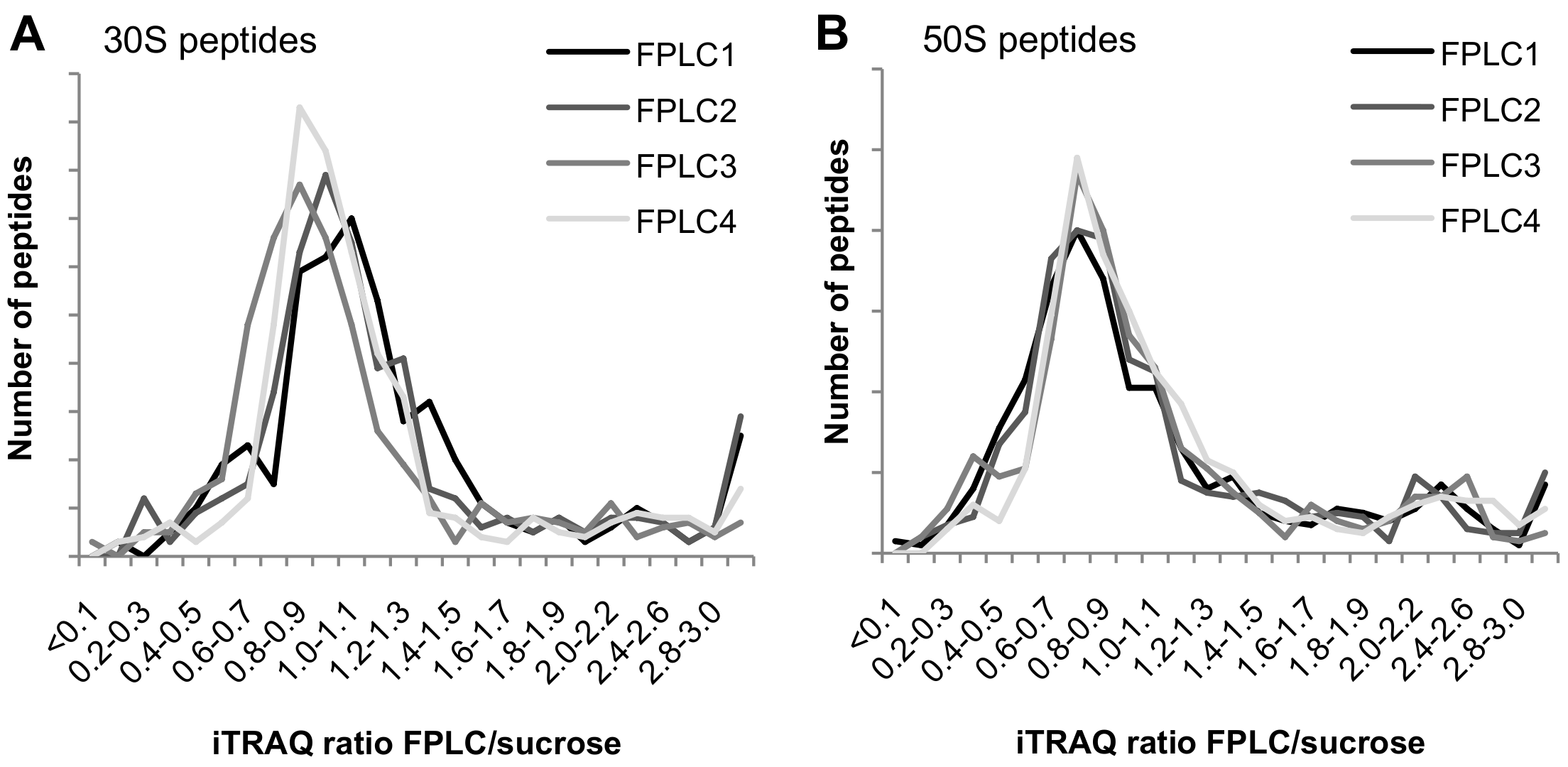

Supplement: Figure S5 — The composition of ribosomes is comparable for repeat FPLC runs and/or repeat samples. Ratios were obtained as described in Fig. S3. FPLC1 and FPLC2 are repeat runs of the same biological sample – actively growing Msm wild type. FPLC3 and FPLC4 were obtained from an independent sample of actively growing Msm wild type. Ratios for 30S peptides (A) and 50S peptides (B) are shown. (TIF) [file pone.0016273.s005.tif]
